# Reviews and Book Notices

**Published:** 1881-10

**Authors:** 


### Reviews and Book Notices.

ARTICLE XIV.—ON THE ANTAGONISM BETWEEN MEDICINES AND BETWEEN REMEDIES AND DISEASES. Being the Cartwright Lectures for the year 1880. By Roberts Bartholow, M.A., M.D., LL.D., Professor of Materia Medica and General Therapeutics in the Jefferson Medical College of Philadelphia; Author of a "Treatise on Materia Medica and Therapeutics," of a "Treatise on the Practice of Medicine," etc. 8vo, cloth, pp. 122. $1.25. New York: D. Appleton & Co. 1881. Chicago: Jansen, McClurg & Co.

It has long been a received principle in medicine that the derangements caused by disease may be opposed and thereby made to disappear by other derangements caused by medicinal substances. Physiological antagonism means an opposition of action of poisonous medicinal agents, in that the effect of the one may be exactly counterbalanced by the effects of the other. But "such completeness of opposing action is as rare as exact similitude in remedies acting in the same way." The Hippocratic aphorism, "in general, diseases are cured by their contraries," remains true. The author then explains the doctrine of similars. "A little consideration must, I think, tend to the conclusion that, when a remedy acts in a similar manner to a disease, there must be an antagonism between the force of the remedy and the momentum acquired by the disease. If the actions were the same, the result of the combined impression would be an increase of the disturbance."

He establishes thus his theory on the basis of a general principle propounded by Herbert Spencer, that the result of the various natural forces acting together is rhythm in action. Bartholow had read Spencer before writing the following: "I am not prepared to say, though I would remark that facts are not an invention of the mind, but have their reality in nature, only awaiting our investigation." Bartholow says: "In the medulla oblongata is situated a center of extensive reflex sensibility, and above it is an inhibitory center of reflex movements." The inference is that antagonistic remedies act on each of these respectively. "In the cardiac and respiratory mechanism we have admirable illustration of opposing forces producing order and rhythm. The movements of the vessels are regulated by a vaso-motor center in the medulla," etc.

That rhythm is the law, however, must not be considered a truism, for, as Bartholow intimates a few lines further, whenever forces interfere in equal proportions they cease to be perceptible, so that only the rhythmical action strikes our senses and thus leads us to think that forces in action are always rhythmical. The perusal of the first lecture will repay any anxious student of philosophy and nature, and leave him convinced that the treatment of disease mostly consists in destroying a natural force (the disease) by another natural force (the remedy or medicinal agent.) The investigation of recorded cases of poisoning by opium, in which belladonna was given as an antidote, is masterly and does honor to the author. A practical point derived from the antagonism of morphia and atropia is their efficiency in preventing any bad result from either drug when given together, while their beneficial effect is increased.

The principle of physiological antagonism is destined to cause an important reform in therapeutics, and any one perusing the book under consideration will get many hints which may prove very valuable in practice; although some of them are startling ideas, they are the more reassuring since they come from Bartholow, and have been, most of them, the result of his careful investigations. Among these, the danger of administering chloral hydrate together with bromide of potassium, and chloral and morphine in full doses, is mentioned. The author claims, however, that an hypodermic injection of morphia, before administering an anæsthetic, is a valuable precaution. Strychnia in paralysis and in phthisis, belladonna in phthisis, and strychnia with ergot in hæmorrhages are all highly recommended. But although comparatively small, this treatise on antagonism contains so many items of information that one could not review more than a few of them, and we must state that a large part of it is concerned with physiological antidotes to vegetable poisons,—a series of facts which every physician must take knowledge of, as it would be considered a criminal neglect not to know the value of belladonna in opium poisoning, etc. It is evident that every student and practitioner of medicine needs this book, and that so much knowledge in so small a compass is very unusual in medicine. H. D. V.

ARTICLE XV.—ON THE BILE, JAUNDICE, AND BILIOUS DISEASES. By J. Wickham Legg, F.R.C.P., Lond.; Assistant Physician to St. Bartholomew's Hospital, and Lecturer on Pathological Anatomy in the Medical School. 8vo, cloth, pp. 720. New York: D. Appleton & Co. 1880.

It will be seen from the title that this is a contribution to physiology and to chemistry, as well as to the practice of medicine, though given mostly to the latter. The subject is a vast one; the author had to follow untrodden paths at times, and the whole necessitated a prodigious amount of experiments. As the fanatical and ignorant act against vivisection had just been passed in the British Parliament, Dr. Legg addressed himself to the French Brotherhood and conducted many of his investigations at the Lyons laboratory, one of the best furnished in Europe. The author expresses himself very bitterly on this subject, calls the agitation a senseless outcry, and adds: "For whatever imperfections there may be in the method followed, the reader must blame those Manichæan teachings, which, harmful as they are, are almost as old as the human race itself, and the germs of which seem to exist everywhere, ready to burst into a fresh life, even amongst those who claim to be most othordox and sincere in their morals and belief."

The author's experiments with the bile of various animals resulted in the fact that its components vary in quantity and quality according to the species. The bile of salt-water fish—the cod, and the turbot, for instance—contains chiefly taurocholate of potash, while the bile of fresh-water fish held much more soda. As a whole these experiments do not seem very satisfactory, and they will not benefit students who need better sifted material. In "Summary of Knowledge as to the Physiology of the Bile," we meet with the statement that "it is uncertain if the liver be a mere filter, or if it secrete the bile itself, although the general belief of physiologists is in favor of the latter." "Whether the breaking down of the tissues, or the splitting up of the peptones from the food furnish the bile acids is not known." "During fasting the amount of bile is much decreased. Purging causes a decrease in amount of bile secreted. Vomiting is followed by an increased expulsion of bile; so also is muscular exercise."

Chapters X to XIX comprise probably the best treatise ever written on the subject of jaundice. Although this is more a symptom than a disease in itself, still any reader will admit after perusing these 188 pages that the subject is better treated in this manner, and rendered much more intelligible to students and even to practitioners.

The following remedies are valuable in the treatment of jaundice: Ferruginous, citrates, acetates of sodium and potassium, mineral waters, lemon juice, acids, both mineral and vegetable.

Chapters XIX to XXIII are devoted to acute yellow atrophy, or icterus gravis. As to its treatment, the author recommends first, a smart mercurial purge, followed by sulphate of magnesia, then large doses of quinine, with an admixture of the mineral acids; locally, a large, warm linseed poultice over the liver. Jaundice after poisoning by phosphorus, copper, mercury, arsenic, antimony, etc., is also described; and so the jaundice which often complicates fevers.

Chapter XXIX and last, treats of bilious diseases, in fourteen pages. This is less than the title of the book suggested. Most fevers come under that head in Hippocrates and Galen's writings. According to Stoll they are troubles of digestion, as diarrhœa, etc. Also, other writers bring under this head a decreased bile secretion.

"Here in England, a bilious attack means vomiting, with headache. If the word bilious means a gastric catarrh, there are still objections to its use."

This book, so far as the matters treated go, will remain a valuable contribution to the study of bile and the treatment of jaundice, and we would recommend every practitioner to get a copy, even if it were only for making references when treating icterus neonatorum, or acute yellow atrophy. H. D. V.

ARTICLE XVI.—MEDICAL ELECTRICITY: A Practical Treatise on the Applications of Electricity to Medicine and Surgery. By Roberts Bartholow, A.M., M.D., LL.D., Professor of Materia Medica and General Therapeutics in the Jefferson Medical College of Philadelphia, etc. Author of "A Practical Treatise on Materia Medica and Therapeutics," and of "A Treatise on the Practice of Medicine," etc. With ninety-six illustrations. 8vo, cloth, pp. 262. $2.50. Philadelphia: Henry C. Lea's Son & Co., 1881. Chicago: Jansen, McClurg & Co.

This comes up to the standard of the author's other works, already so well known. Dr. Bartholow represents rationalism in medicine, and every line dropped from his pen is the expression of sound theories and practical facts. One should not expect to find electricity in medicine so highly commended as in Beard and Rockwell's large work, for this author is not a specialist, and would not perhaps deny the following statement of a contemporary: "Electricity has not as yet attained a very wide range of usefulness in medicine or surgery. The cost, the inconvenience, and the lack of knowledge have had something to do with this. And besides, the intrinsic value of the agent is undoubtedly less than was at first supposed and hoped." Yet electricity in the hands of the author has been very serviceable in the treatment of paralysis, rheumatism, many nervous diseases, and in constitutional diseases also; electricity at times acting as a tonic, and also as an alterative.

This book is well printed, the type is large and clear, and the contents are about all that a busy practitioner has time to peruse. It is also well adapted to students of medicine, and will sufficiently supplement their knowledge of electro-physics, of which so few of them know anything, resembling their preceptors in this. Although some enthusiastic minds once cherished the idea that electricity was the source of all other forces, that it was the soul of nature, and consequently a promoter of life, these theories have faded away, and medical electricity received a great stroke at the hand of that writer who lately proved that it was mainly a paralyzing agent. However, electricity remains one of the surest ways to influence the mind, and thus exercise a beneficial effect on the body. H. D. V.

ARTICLE XVII.—HERNIA, STRANGULATED AND REDUCIBLE. With Cure by Subcutaneous Injections, together with Suggested and Improved Methods for Kelotomy. Also an Appendix, giving a short account of various Surgical Instruments. By Joseph H. Warren, M.D., Member of American Medical Association, etc. 8vo, cloth, pp. 280. $3.00. With illustrations. Boston: Charles N. Thomas, 215 Tremont street. London: Sampson, Low, Marston, Searle, and Rivington. 1881.

This is certainly a *multum in parvo*, and that little consists in the author's operation, which is detailed in seventy-three pages, and is, as the profession is aware, a very promising treatment. A few quotations will convey the author's idea. A special syringe, invented by Dr. Warren, should be used, as no other hypodermic needle than his will answer the purpose. The legs of the patient being drawn up and the muscles relaxed, the hernia should be returned, then, "we pass the left middle finger up the spermatic canal until we come to the inguinal ring, and by slightly raising the end of the middle finger as above mentioned, the same is felt by the fore finger, which also helps us to indicate the exact point, and guide to insert the point of the instrument. Having ascertained that the ring is well open, and free from attachments or adhesions to the returned sac, we begin to insert the needle at the lower portion of the ring, where we feel its edges through the abdominal parietes. The needle should always enter this lower portion of the ring, as in passing obliquely upwards and backwards it is less likely to wound either column of the internal ring. Great care should be taken in inserting it through the integuments and superficial fascia, so as not to wound the external pillar, but to enter the canal at once. The needle then should never be passed in a perpendicular direction, as there is thus danger of wounding the spermatic cord, but it should receive the necessary obliquity as soon as we feel that it has passed through the integuments. We can diagnose the position of the needle when first entering, by passing the left fore and little finger up with the invaginated scrotum upon it. When we have passed the needle though the integuments, we begin to open the valve and slowly push the needle in the direction already indicated. As the needle is thus inserted, it revolves and injects the fluid in sufficient quantities to cover well the external and internal rings. The needle is now slowly withdrawn, still injecting fluid in its backward motion. As soon as the needle is withdrawn pressure is made with the end of the fingers over the wound and rings for five or ten minutes, until the smarting and throbbing pain subsides."

A compress is now applied over the inguinal canal, and should be kept wet with cold water. The patient should stay in bed for a few days, and opiates should be administered during that time. Dr. Warren claims that all cases do well, and that was also the statement of the late Dr. Heaton, who used an infusion of white oak bark for an injecting fluid and a blunt hypodermic needle. Any one desiring to employ this treatment of hernia should read Dr. Warren's book and procure his instruments.

The reviewer believes in the correctness of the author's statements, yet would add that notwithstanding the wide advertisement which this method had in Europe as well as in America, he has not come across any statement in the medical press of France, England, Canada, nor the United States, to corroborate these wonderful cures. Of course this method of treatment is so new that positive results could not be well ascertained, although the seemingly harmlessness of the operation should open a wide field to enterprising surgeons, and it is time that it should be rescued from the hands of quacks. H. D. V.

